# Nuclear Condensates of WW Domain‐Containing Adaptor With Coiled‐Coil Regulate Mitophagy via Alternative Splicing

**DOI:** 10.1002/advs.202406759

**Published:** 2025-01-22

**Authors:** Jiahe Wang, Yi Fan, Guowen Luo, Liang Xiong, Lijie Wang, Zhuoxuan Wu, Jiayi Wang, Zhengying Peng, Clifford J Rosen, Kefeng Lu, Junjun Jing, Quan Yuan, Zhenwei Zhang, Chenchen Zhou

**Affiliations:** ^1^ State Key Laboratory of Oral Diseases and National Clinical Research Center for Oral Diseases West China Hospital of Stomatology Sichuan University Chengdu 610041 China; ^2^ State Key Laboratory of Biotherapy and Department of Rheumatology and Immunology West China Hospital Sichuan University Chengdu 610041 China; ^3^ Maine Medical Center Research Institute Scarborough ME 04074 USA; ^4^ Department of Neurosurgery State Key Laboratory of Biotherapy West China Hospital Sichuan University and The Research Units of West China Chinese Academy of Medical Sciences Chengdu 610041 China

**Keywords:** mitophagy, nuclear speckles, phase‐separated biomolecular condensates, pre‐mRNA splicing, snRNP, WAC

## Abstract

Biomolecular condensates segregate nuclei into discrete regions, facilitating the execution of distinct biological functions. Here, it is identified that the WW domain containing adaptor with coiled‐coil (WAC) is localized to nuclear speckles via its WW domain and plays a pivotal role in regulating alternative splicing through the formation of biomolecular condensates via its C‐terminal coiled‐coil (CC) domain. WAC acts as a scaffold protein and facilitates the integration of RNA‐binding motif 12 (RBM12) into nuclear speckles, where RBM12 potentially interacts with the spliceosomal U5 small nuclear ribonucleoprotein (snRNP). Importantly, knockdown of RBM12, or deletion of the WAC CC domain led to altered splicing outcomes, resulting in an elevated level of BECN1‐S, the short splice variant of BECN1 that is shown to upregulate mitophagy. Thus, the findings reveal a previously unrecognized mechanism for the nuclear regulation of mitochondrial function through liquid–liquid phase separation (LLPS) and provide insights into the pathogenesis of WAC‐related disorders.

## Graphical abstract

WW domain containing adaptor with coiled‐coil (WAC) forms nuclear condensates and regulates alternative splicing through its coiled‐coil domain. WAC scaffolds RBM12, potentially interacting with U5 snRNP. Alterations in WAC affect alternative splicing and elevating BECN1‐S expression which leads to hyperactivated mitophagy.

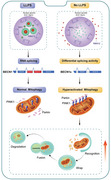

## 1 Introduction

In the intricate milieu of the eukaryotic cell nucleus, the organization of biological functions into distinct spatial domains without the confinement of membranes represents a paradigm shift in our understanding of cellular compartmentalization. Membraneless organelles (MLOs), such as nuclear speckles, Cajal bodies, and promyelocytic leukemia (PML) nuclear bodies, epitomize this phenomenon, serving as focal points for the segregation and execution of nuclear functions.^[^ ^1^ ^]^ Central to the formation of these organelles is the process of liquid‐liquid phase separation (LLPS), a biophysical mechanism that separates cellular components into coexisting liquid phases through multivalent interactions between proteins and nucleic acids. This emergent mechanism underscores a novel paradigm for cellular organization, facilitating the dynamic yet orderly arrangement of physiological processes. By leveraging the intrinsic properties of biomolecules, LLPS facilitates a reversible and adaptable structuring of the intracellular environment, thereby enhancing cellular efficiency and responsiveness to physiological stimuli. This dynamic compartmentalization offers a nuanced explanation of how cells prioritize and regulate complex biochemical pathways, ensuring fidelity in the execution of genomic instructions and adaptive responses to environmental changes.

Nuclear speckles are essential MLOs for pre‐mRNA processing. They serve not only as storage sites for splicing factors but also regulate transcription and mRNA maturation.^[^ ^2^ ^]^ The composition of nuclear speckles includes the spliceosome, SR family proteins, heterogeneous nuclear ribonucleoproteins (hnRNPs), and other unidentified proteins.^[^ ^3^ ^]^ The spliceosome is composed of U1, U2, U4/U6, and U5 snRNP and other non‐snRNP splicing factors and is recruited to pre‐mRNAs for splicing.^[^ ^4^ ^]^ Previous studies on dynamic MLOs have traced the dynamic motility events of nuclear speckles using time‐lapse fluorescence imaging.^[^ ^5^, ^6^ ^]^ Many proteins within nuclear speckles, including the splicing factor SRRM2,^[^ ^7^ ^]^ and SON,^[^ ^8^ ^]^ have been shown to form nuclear condensates by undergoing LLPS. These proteins act as essential scaffolds, holding the proteins in the nuclear speckles together.^[^ ^9^ ^]^ Interestingly, the LLPS dynamics of nuclear speckles follow a cell‐autonomous 12‐h rhythm controlled by the XBP1s‐SON axis.^[^ ^8^ ^]^ Many splicing regulators, including SFSR1,^[^ ^10^ ^]^ AKAP95^[^ ^11^ ^]^ and WASP,^[^ ^12^ ^]^ also regulate splicing by undergoing LLPS, with impaired condensate formation weakening the activity of splicing regulation.

WW domain‐containing adaptor with coiled‐coil (WAC) is a multifunctional adaptor protein consisting of two well‐characterized domains, a WW domain at the N‐terminus and a coiled‐coil (CC) domain at the C‐terminus. WAC is a regulator of multiple cellular functions, including histone H2B ubiquitination and transcription^[^ ^13^ ^]^ and timely entry into mitosis.^[^ ^14^ ^]^ In addition, WAC‐mediated H2B ubiquitination regulates plasma cell differentiation.^[^ ^15^ ^]^ Mutations in the WAC gene cause DeSanto‐Shinawi syndrome, a recognizable genetic syndrome characterized by developmental delay, hypotonia, and dysmorphic features,^[^ ^16^ ^]^ which suggests that WAC plays essential roles in the skeletal and nervous systems. Early studies indicated that WAC highly colocalizes with SC35, a nuclear speckle marker.^[^ ^17^ ^]^ Interestingly, the yeast ortholog of human WAC, Lge1, acts as a scaffold protein to promote the ubiquitination of H2B by interaction with Bre1 (the yeast ortholog of the human ubiquitin ligase RNF20/RNF40) via LLPS.^[^ ^18^ ^]^ However, whether WAC functions in regulating pre‐mRNA splicing is largely unknown.

Here, we reveal that WAC acts as a scaffold protein in the nuclear speckle and can form biomolecular condensates to regulate pre‐mRNA splicing. Our findings demonstrate that WAC undergoes LLPS through its C‐terminal CC domain, with the resulting LLPS structures localizing to nuclear speckles via its N‐terminal WW domain. Acting as a scaffold protein, WAC binds to and induces co‐phase separation with the client protein RBM12, which interacts with the spliceosomal U5 snRNP. Disruption of WAC‐mediated LLPS results in altered splicing outcomes of mitophagy genes and affects mitochondrial homeostasis. Our findings shed new light on the pathogenesis of DeSanto‐Shinawi syndrome and may broaden our understanding of nuclear condensate‐mediated regulation of AS.

## 2 Results

### 2.1 WAC Localizes to Nuclear Speckles and Regulates AS

We first examined the spatial relationship between WAC and nuclear speckles (NSs). Immunofluorescence assays were employed to analyze the cellular localization of WAC and the NS markers SON and SC35 in HeLa cells.

**Figure 1:**
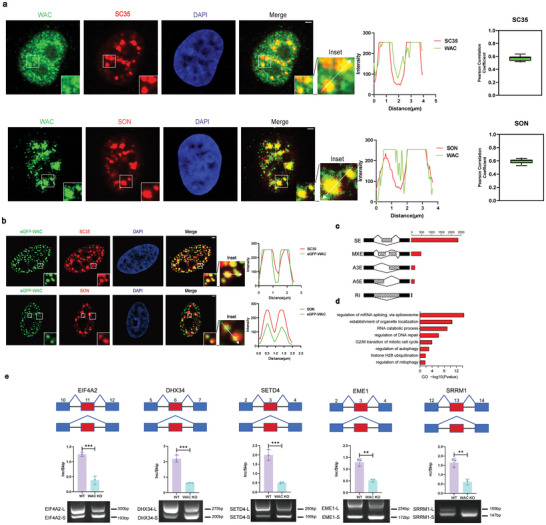
WAC colocalizes with nuclear speckles and regulates alternative splicing. a) Immunofluorescence assays were conducted to visualize the colocalization of endogenous WAC with the endogenous nuclear speckle markers SC35 and SON. The Pearson correlation coefficient was used to assess the overlap between each fluorescence signal in the nucleus region. Green boxes contain the 25th to 75th percentiles of the dataset (n = 15). Black whiskers mark the 10th and 90th percentiles. Outliers are marked with black dots. b) Immunofluorescence assays were conducted to visualize the colocalization of eGFP‐tagged WAC with the endogenous nuclear speckle markers SC35 and SON. Scale bar = 2 µm. c) Quantification of DASEs affected by WAC. d) Gene ontology of WAC‐regulated AS targets. e) Validation of randomly selected AS events by semi‐quantitative RT‐PCR and quantification of relative splicing efficiency of representative mRNAs upon WAC knockout in HeLa cells. The relative Inc/Skip (inclusion band/exclusion band) ratio was plotted (n = 3, Student's t‐test). ^*^ *p* < 0.05; ^**^ *p* < 0.01; ^***^ *p* < 0.001 ^****^ *p* < 0.0001. The results are from more than three independent experiments. Values are mean ± SD.

In HeLa cells with endogenous levels of WAC and those transfected with eGFP‐WAC, we observed strong colocalization of WAC with the NS markers SON and SC35 (**Figure** **1**a,b). Furthermore, markers for other nuclear membraneless organelles (MLOs), such as NPM1 for the nucleolus and coilin for Cajal bodies, did not colocalize with WAC (Extended Data Figure 1a,b). These findings suggest that WAC is a specific constituent of nuclear speckles and not a general component of other nuclear MLOs.

To further investigate the role of WAC in splicing regulation, we generated WAC‐knockout HeLa cell lines using CRISPR/Cas9 technology (Extended Data Figure 1c) and confirmed the knockout efficiency by immunoblotting (Extended Data Figure 1d). RNA sequencing (RNA‐seq) experiments were performed to assess the impact of WAC on alternative splicing. Analysis of differential alternative splicing events (DASEs) indicated that WAC knockout resulted in alterations of thousands of mRNAs.

The various types of DASEs regulated by WAC (percent spliced in (PSI)≥0.15, P<0.05, FDR<0.05), in descending order of prevalence, were: skipped exons (SEs), mutually exclusive exons (MXEs), alternative 3′ splice site exons (A3Es), alternative 5′ splice site exons (A5Es), and retained introns (RIs) (Figure 1c). Gene Ontology (GO) analysis of the biological processes associated with WAC‐regulated ASEs identified genes involved in multiple pathways, including “regulation of mRNA splicing via spliceosome,” “establishment of organelle localization,” “regulation of autophagy”, and “regulation of mitophagy” (Figure 1d). Furthermore, we performed RT‐PCR experiments to validate these ASEs regulated by WAC. We randomly selected and examined the alternative splicing of several representative mRNA targets (including EIF4A2, DHX34, SETD4, EME1, and SRRM1) and confirmed that WAC mediated their proper splicing (Figure 1e).

Interestingly, we found that both endogenous WAC and overexpressed WAC‐eGFP fusion proteins exhibited properties resembling those of biomolecular condensates. We next sought to investigate whether WAC regulates alternative splicing through the formation of nuclear condensates.

### 2.2 WAC Forms Biomolecular Condensates In Vivo and In Vitro

**Figure 2:**
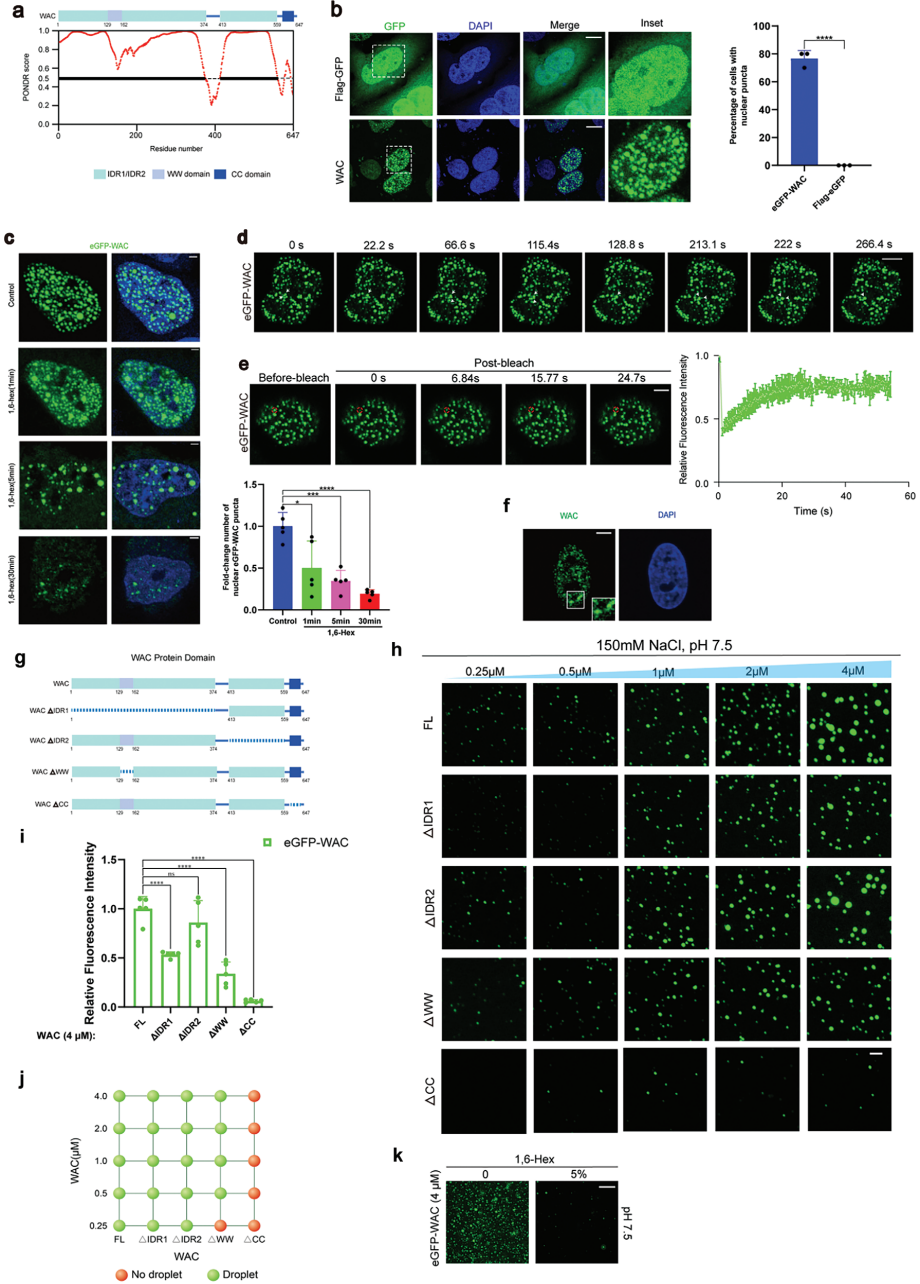
WAC forms biomolecular condensates in vivo and in vitro. a) PONDR (www.pondr.com) prediction of intrinsically disordered regions (IDRs) of WAC domain architectures is shown. b) Representative images of HeLa cells transfected with eGFP‐WAC and eGFP plasmids. The percentage of nuclear condensates formed in cells expressing fluorescent proteins from three independent experiments is shown (n = 15, Student's t‐test). Scale bar = 10 µm. c) Representative images showing the effects of eGFP‐WAC plasmid transfection in HeLa cells with or without 1,6‐hexanediol (1,6‐HD) treatment (5%, 1, 5, and 30 min). The number of condensates formed per cell with or without 1,6‐HD treatment was quantified in three independent experiments (n = 15, Student's t‐test). Scale bar = 2 µm. d) Images of two fusion events (arrowheads) among WAC condensates. See Video S1 (Supporting Information). e) Representative time‐lapse images with magnified insets showing the fluorescence recovery after photobleaching (FRAP) of eGFP‐WAC puncta. The curve on the right shows the relative quantification of the fluorescence signal recorded pre‐ and postbleach (n = 6). Scale bar = 5 µm. f) Immunofluorescence assays were conducted to inspect the nuclear condensation of endogenous WAC. Scale bar = 2 µm. g) Diagram of domain mapping of four truncated versions of human WAC mutants. h) In vitro condensate formation of purified full‐length and 4 truncated WAC fusion proteins at various protein concentrations (0.25, 0.5, 1, 2, or 4 µm). Scale bar =5 µm. i) The quantification of the droplets shown in Figure 2h. Droplets in each group were quantified (n = 5, one‐way ANOVA). j) Phase diagram of Figure 2h. k) In vitro condensate formation of purified full‐length WAC fusion proteins with or without 1,6‐hexanediol (1,6‐HD) treatment. Scale bar = 10 µm. ^*^ *p* < 0.05; ^**^ *p* < 0.01; ^***^ *p* < 0.001; ^****^ *p* < 0.0001. The results are from more than three independent experiments. Values are mean ± SD.

Given the tendency of proteins with intrinsically disordered regions (IDRs) to form biomolecular condensates,^[^ ^19^ ^]^ we used the Predictor of Natural Disordered Regions (PONDR) to identify IDRs within the WAC protein. Our analysis revealed two major segments, IDR1 and IDR2, that are highly disordered. In addition to IDR1 and IDR2, the WAC protein contains a WW domain and a CC domain (**Figure** **2**a). In contrast to eGFP alone, phase‐separated eGFP‐WAC puncta formed in the nucleus following transfection, indicating a distinct localization pattern in the cells (Figure 2b). The alcohol 1,6‐hexanediol, widely used in LLPS studies, has the capacity to dissolve phase‐separated condensates by interfering with hydropathic interactions within biomacromolecules.^[^ ^20^ ^]^ After the addition of 1,6‐hexanediol, the number of eGFP‐WAC nuclear puncta of eGFP‐WAC diminished over time (Figure 2c). Live‐cell imaging of HeLa cells revealed fusion events when two eGFP‐WAC puncta contacted each other (Figure 2d; Video S1, Supporting Information). In addition, fluorescence recovery photobleaching (FRAP) analysis revealed that the eGFP‐WAC puncta quickly recovered after several seconds (Figure 2e; Video S2, Supporting Information). This indicates that these condensates possess dynamic exchange and recovery capabilities within the cell. Importantly, endogenous WAC also formed nuclear puncta in the cells (Figure 2f). This evidence confirmed that the WAC protein forms biomolecular condensates that exhibit specific localization within the nucleus and dynamic exchange and recovery properties. Furthermore, 5% 1,6‐hexanediol was capable of dissolving eGFP‐WAC condensates in vitro (Figure 2j). The in vitro condensate formation of eGFP‐WAC reached saturation near physiological NaCl concentrations (150 mm), with increased NaCl concentrations resulting in weakened condensate formation (Extended Data Figure 2c–e). Diverse pH levels were found to have varying impacts on droplet morphology, with condensates formed by eGFP‐WAC appearing smaller in size and more spherical near a physiological pH of 7.5. This property is hypothesized to ensure the fluidity of nuclear condensates, facilitating their physiological function (Extended Data Figure 2f,g).

### 2.3 The Coiled‐Coil Domain of WAC Drives Condensates Formation

To further elucidate how the domains within WAC influence its LLPS capability, we engineered four truncated eGFP‐WAC variants (ΔIDR1, ΔIDR2, ΔWW, and ΔCC) along with the full‐length eGFP‐WAC, and performed in vitro purification of each protein variant (Figure 2g and Extended Data Figure 2a,b). Under conditions of 150 mm NaCl and pH 7.5 (close to physiological conditions), purified full‐length eGFP‐WAC protein exhibited condensate formation at a final concentration of 4 µm. The truncated variants showed diminished condensate formation capabilities to varying extents. Notably, condensate formation by the purified eGFP‐WACΔCC protein was scarcely observed in vitro (Figure 2h–j).

**Figure 3:**
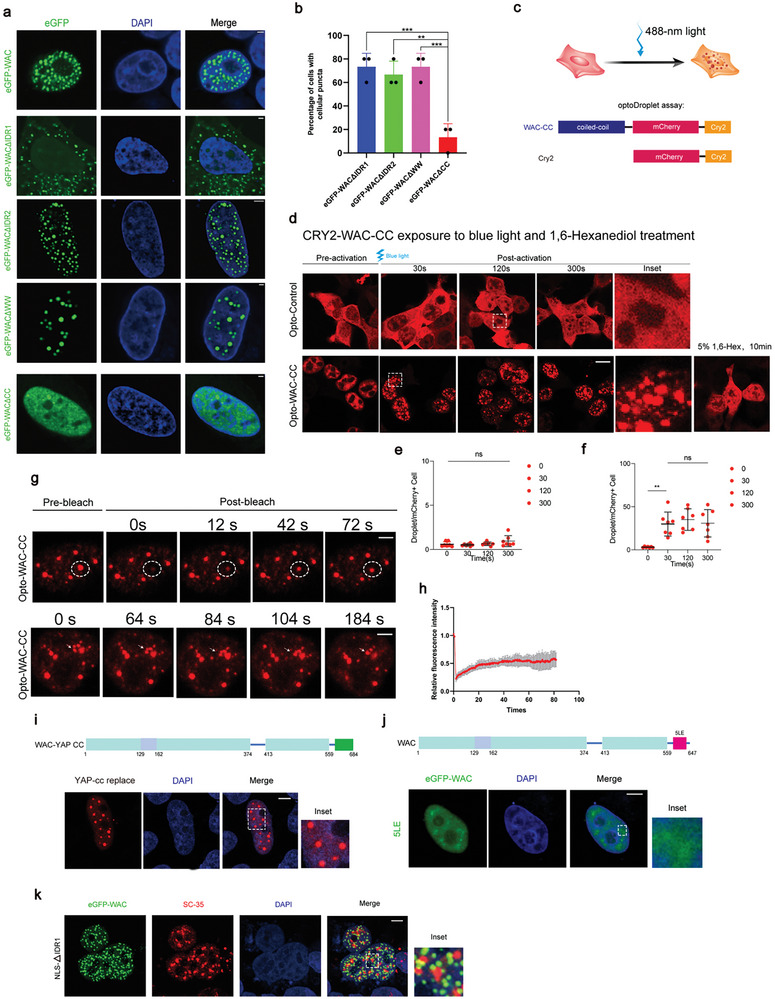
The coiled‐coil domain of WAC drives condensate formation. a) Fluorescence images of HeLa cells transfected with full‐length and 4 truncated versions of WAC with eGFP. Scale bar = 2µm. b) The percentage of cells that displayed cellular puncta is shown on the right. The percentage of cellular condensates expressing fluorescent proteins from three independent experiments is shown (n = 15, one‐way ANOVA). Scale bar = 2 µm. c) Experimental workflow of light‐induced optoDroplet formation of Cry2‐mCherry‐WAC. Diagram of domain mapping of the plasmid used in the optoDroplet assay. d) Representative images of opto‐Droplet formation before and after light induction are shown. Scale bar = 10 µm. e‐f) Quantification of the droplets formed by Opto‐Control or Opto‐WAC before and after light induction is shown. Seven transfected cells in each group were randomly selected and quantified (n = 7, one‐way ANOVA). g‐h) Representative time‐lapse images with magnified insets showing the FRAP recovery of puncta formed by Cry2‐mCherry‐WAC or Cry2‐mCherry. The curve on the right shows the relative quantification of the fluorescence signal recorded pre‐ and postbleach (n = 3). Scale bar = 5 µm. i‐j) Diagram of 2 truncated domain maps of human WAC mutants. Immunofluorescence images of HeLa cells transfected with eGFP‐WAC‐YAP CC and eGFP‐WAC‐5LE with SC35. Scale bar = 2 µm. k) Immunofluorescence images of HeLa cells transfected with eGFP‐WACΔIDR1‐NLS and SC35. Scale bar = 5 µm. ^*^ *p* < 0.05; ^**^ *p* < 0.01; ^***^ *p* < 0.001; ^****^ *p* < 0.0001. The results are from more than three independent experiments. Values are mean ± SD.

Consistent with the in vitro phenotypes, in vivo experiments under identical transfection conditions with two constructs (lacking the WW domain or the IDR2 domain) showed nuclear puncta resembling those observed with full‐length WAC (**Figure** **3**a,b). Intriguingly, cells transfected with eGFP‐WACΔIDR1 displayed puncta outside the nucleus (Figure 3a). We speculate that the IDR1 sequence may contain a nuclear localization signal (NLS) for the nuclear localization of WAC. Indeed, introducing an additional NLS sequence in eGFP‐WACΔIDR1 restored nuclear puncta and restored colocalization with nuclear speckles (Figure 3k). Remarkably, deletion of the CC domain of WAC led to the dispersion of nuclear WAC puncta (Figure 3a,b). Taken together, our results suggest that the CC domain is essential for the formation of WAC condensates, while the IDR1 domain is responsible for its nuclear localization.

To further examine whether the CC domain of WAC can effectively induce phase separation, we fused the CC domain with mCherry‐Cry2 (Figure 3c) and assessed condensate formation using the optoDroplet method.^[^ ^21^ ^]^ After stimulation with blue light, the CC‐mCherry‐Cry2 fusion protein formed condensates, and the addition of 1,6‐hexanediol dissolved these puncta (Figure 3d–f). Subsequently, we discovered that the Opto‐WAC‐CC droplets within the nucleus could fuse and exchange materials with the surrounding nucleoplasm (Figure 3g). YAP is a transcriptional activator that undergoes phase separation driven by its CC domain.^[^ ^22^ ^]^ We replaced the CC domain of WAC with that of YAP and observed nuclear puncta with similar morphology (Figure 3i). Moreover, we mutated five leucines in the CC domain into five glutamic acids (eGFP‐WAC‐5LE) to reverse the hydrophilicity, which completely blocked nuclear condensate formation (Figure 3j). In summary, WAC phase separation is initiated by CC‐mediated hydrophobic interactions.

### 2.4 WAC Serves as a Scaffold Protein for RBM12

**Figure 4:**
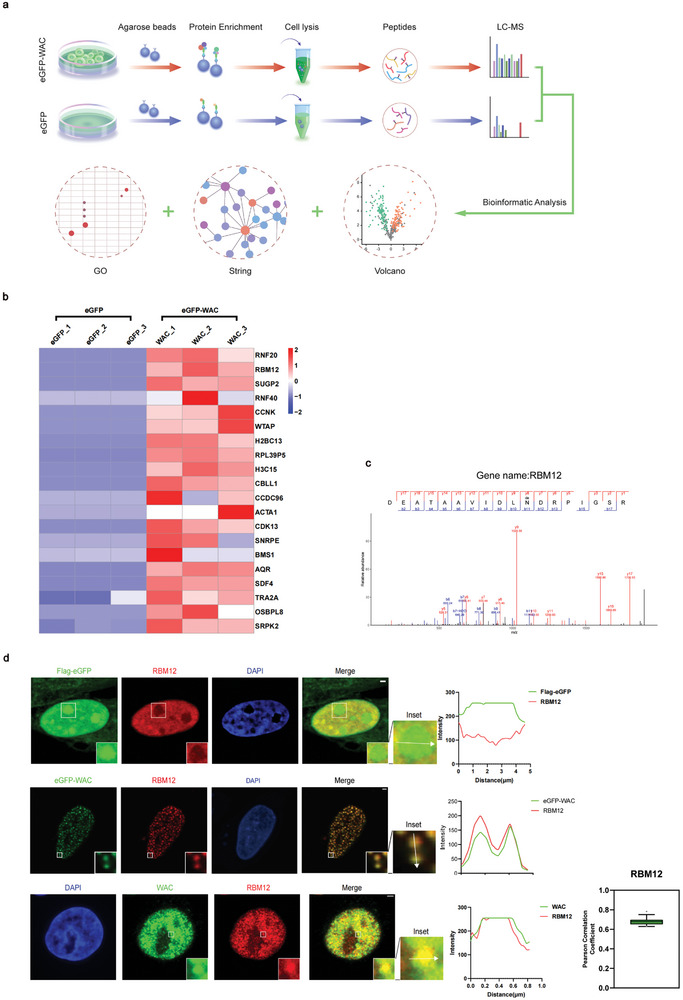
Mass spectrometry identified RBM12 as an interacting protein of eGFP‐WAC. a) A schematic diagram illustrating the immunoprecipitation–mass spectrometry procedure used in this study is shown. b) Heatmap of identified eGFP‐WAC‐interacting proteins. c) Mass spectrometry analysis of eGFP‐WAC immunoprecipitates. Selected peptide hits and unused scores are shown. A higher unused score means more credibility. d) Immunofluorescence assays were conducted to evaluate the colocalization of eGFP tag or eGFP‐tagged WAC and endogenous WAC with endogenous RBM12. The Pearson correlation coefficient was used to assess the overlap between each fluorescence signal in the nucleus region. Green boxes contain the 25th to 75th percentiles of the dataset. Black whiskers mark the 10th and 90th percentiles. Outliers are marked with black dots. Scale bar = 2 µm.

Current research has identified several common features of molecules capable of undergoing phase separation under physiological conditions. Among these are scaffolds and client proteins, with scaffold molecules considered the driving force behind phase separation and proteins participating in droplet formation post‐phase separation termed as client proteins.^[^ ^23^ ^]^ To further elucidate the potential molecular basis of WAC‐related AS, we carried out liquid chromatography (LC)‐tandem mass spectrometry (MS)/MS analysis to identify potential WAC protein–protein interactions (**Figure** **4**a).

GO analysis of WAC‐interacting proteins revealed functional enrichment in Molecular Function, specifically in snRNP and snRNA binding (Extended Data Figure 3a). snRNPs, key components of the spliceosome, play a pivotal role in recognizing splice sites on pre‐mRNA and catalyzing the splicing reaction,^[^ ^24^ ^]^ while snRNA, part of snRNP complexes, is involved in splice site recognition and regulation of the splicing reaction.^[^ ^24^ ^]^ Therefore, the significant enrichment of WAC in snRNP and snRNA binding strongly suggests its involvement in the assembly or function of the spliceosome. We then created a C‐terminal‐tagged eGFP‐WAC knock‐in cell line and utilized Cy3‐labeled probes for the spliceosomal marker U2 snRNA in RNA fluorescence in situ hybridization (FISH) and immunofluorescence experiments. We observed spatial colocalization between endogenously tagged WAC and U2 snRNA, demonstrating the physical proximity of WAC to spliceosomal components within cells (Extended Data Figure 3b,c).

We identified RNF20 and RNF40 as the top candidates for eGFP‐WAC which was consistent with previous findings and confirmed the solidity of this IP/MS analysis. Among all proteins identified as enriched in the eGFP‐WAC group, we focused on those located in the nucleus and detected an enrichment of proteins potentially regulating AS. Among these proteins, RBM12, a nuclear RNA‐binding protein, emerged as one of the top candidates for interaction with eGFP‐WAC (Figure 4b,c). Immunofluorescence revealed that endogenous RBM12 displayed dispersed nuclear expression with week condensation. Upon coexpression with eGFP‐WAC, endogenous RBM12 was incorporated into highly fluidic eGFP‐WAC puncta (Figure 4d). Besides, we observed apparent colocalization between WAC and RBM12 under endogenous level, which indicates an interaction to regulate pre‐mRNA processing at physiological levels (Figure 4d).

### 2.5 Multiphase Dynamics in WAC‐RBM12 Interactions

**Figure 5:**
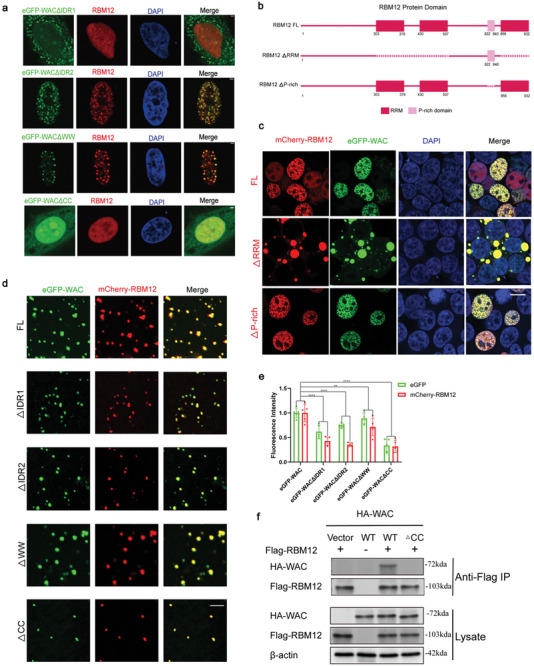
WAC interacts with the client protein RBM12 to cause co‐phase separation. a) Immunofluorescence images of HeLa cells transfected with 4 truncated versions of WAC with eGFP and endogenous RBM12. Nuclei were stained with 4’,6‐diamidino‐2‐phenylindole (DAPI). Scale bar = 2 µm. b) Diagram of 3 truncated domain maps of human RBM12 mutants. Human RBM12 contains 3 RNA recognition motifs (RRMs) and 1 proline‐rich linker region. c) Coexpression of 3 truncated versions of RBM12 with mCherry and eGFP‐WAC. Nuclei were stained with DAPI. Scale bar = 10 µm. d) In vitro condensate formation of purified full‐length or 4 truncated versions of eGFP–WAC (10 µM) mixed with 10 µm WT mCherry–RBM12. Scale bar = 5 µm. e) The quantification of the droplets is shown in Figure 5D. Droplets in each group were quantified (n = 5, two‐way ANOVA). f) The ability of HA‐tagged wild‐type WAC or WACΔCC to interact with Flag–RBM12 was examined using a coimmunoprecipitation assay with anti‐Flag antibodies via immunoprecipitation (IP), followed by western blotting with anti‐HA antibodies (top). The abundance of these proteins in the cell lysates was examined using western blotting (bottom). ^*^ *p* < 0.05; ^**^ *p* < 0.01; ^***^ *p* < 0.001; ^****^ *p* < 0.0001. The results are from more than three independent experiments. Values are mean ± SD.

We next set out to investigate the regions of WAC that are important for the co‐phase separation of WAC and RBM12. After transfecting cells with four truncated eGFP‐WAC constructs (ΔIDR1, ΔIDR2, ΔWW, and ΔCC), we found that deletion of the WAC IDR1 domain prevented RBM12 from forming droplets, as WAC could not be imported into the nucleus. Expression of ΔIDR2, ΔWW, and FL‐eGFP‐WAC enabled RBM12 to participate in the formation of eGFP‐WAC droplets (**Figure** **5**a). However, when the CC domain was deleted(ΔCC), WAC lost its phase separation ability, and RBM12 was dispersed within the nucleus. Immunoprecipitation (IP) analysis also confirmed that deletion of the WAC CC domain abolishes the interaction between HA‐WAC and Flag‐RBM12 (Figure 5f). Next, we purified the full‐length mCherry‐RBM12 protein (Extended Data Figure 3d), and mixed different constructs of eGFP‐WAC (full‐length, ΔIDR1, ΔIDR2, ΔWW, and ΔCC) proteins with the mCherry‐RBM12 protein in vitro. Using confocal microscopy, we observed differing degrees of attenuated condensate formation when the truncated eGFP‐WAC proteins were mixed with mCherry‐RBM12 (Figure 5d,e), while the purified eGFP and mCherry control proteins showed no significant condensate formation (Extended Data Figure 3f). The co‐phase separation ability of eGFP‐WACΔCC with mCherry‐RBM12 was the lowest, with the faintest fluorescence intensity observed in the formed condensates (Figure 5d,e), consistent with the results observed in cells. Thus, our in vivo and in vitro results reveal that WAC functions as a scaffold protein during phase separation to incorporate the client protein RBM12 into droplets (Figure 5a), and that this function is dependent on its CC domain.

Human RBM12 consists of three RNA recognition motifs (RRMs) and one proline‐rich linker region (Figure 5b). To investigate the roles of these regions in co‐phase separation, we constructed plasmids containing truncated mCherry‐RBM12 (ΔRRM and ΔP‐rich) and transfected them into cells. While deletion of the P‐rich region did not affect RBM12 localization nor its co‐phase separation ability with WAC in the nucleus, deletion of RRMs altered the nuclear localization pattern of RBM12, indicating that the RRMs are essential for nuclear localization (Figure 5c, Extended Data Figure 3e). Interestingly, the coexpression of eGFP‐WAC and mCherry‐RBM12 (ΔRRM) revealed that the deletion of RRMs in RBM12 caused the formation of WAC‐RBM12 droplets out of the nucleus (Figure 5c).

In summary, these experiments revealed that WAC functions as a scaffold protein capable of recruiting client proteins, such as RBM12, to specific cellular locations. This regulatory mechanism is underpinned by the multivalent and dynamic interactions between these proteins.

### 2.6 Biomolecular Condensates of WAC Control RBM12 Integration Into Nuclear Speckles

**Figure 6:**
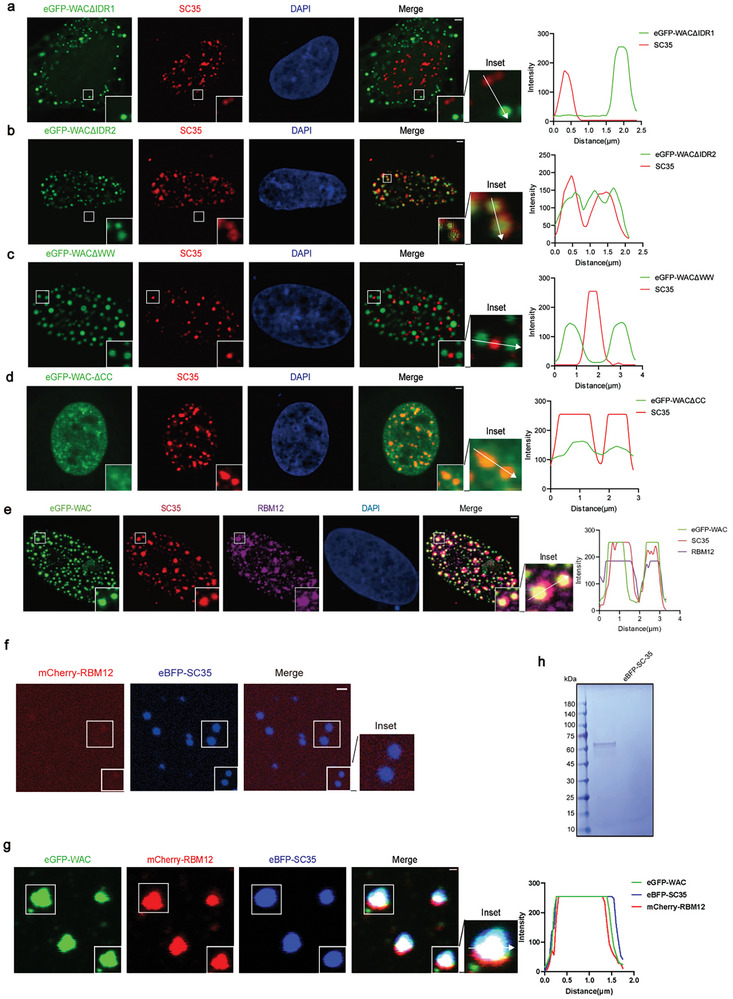
WAC integrates RBM12 into nuclear speckles. a‐d) Immunofluorescence images of HeLa cells transfected with full‐length WAC and 4 truncated versions of WAC with eGFP and endogenous SC35. Nuclei were stained with 4’,6‐diamidino‐2‐phenylindole (DAPI). Scale bar = 2 µm. e) Immunofluorescence images of HeLa cells transfected with full‐length and 4 truncated versions of WAC with eGFP and both endogenous SC35 and RBM12. Nuclei were stained with DAPI. Scale bar = 2µm. f) In vitro cophase separation of mCherry‐RBM12 with eBFP‐SC35. The reactions were performed in 150 mm NaCl (pH 7.0). g) In vitro cophase separation of eGFP‐WAC with mCherry‐RBM12 and eBFP‐SC35. The reactions were performed in 150 mm NaCl (pH 7.0). h) Coomassie blue staining of SDS‒PAGE gel showing the purity of SC35 fused to eBFP.

Subsequently, to identify the domain that is critical for the nuclear speckle localization of WAC, we transfected cells with plasmids encoding truncated WAC (ΔIDR1, ΔIDR2, ΔWW, and ΔCC) fused to eGFP to explore the relationship between their distribution and that of SC35. After transfection, WAC colocalized with SC35 in cells expressing the ΔIDR2 truncation (**Figure** **6** a–d) However, in cells expressing the ΔIDR1 truncation, WAC was excluded from the nucleus and thus could not colocalize with SC35 in nuclear speckles (Figure 6a–d). WACΔCC exhibited dispersed nuclear expression, while eGFP‐WACΔWW puncta exhibited a morphology similar to that of nuclear condensates but did not colocalize with SC35 (Figure 6a–d). These results suggested that the WW domain of WAC likely determines its localization to nuclear speckles.

Further, we purified the eBFP‐SC35 protein and mixed it in vitro with full‐length eGFP‐WAC and full‐length mCherry‐RBM12 proteins. Confocal microscopy showed that this protein mixture formed condensates via co‐phase separation, while the mixture of mCherry‐RBM12 and eBFP‐SC35 alone could not form co‐phase condensates (Figure 6f,g). This in vitro observation is consistent with in vivo experiments in which endogenous RBM12 was incorporated into nuclear speckles by eGFP‐WAC‐formed phase‐separated condensates (Figure 6e). These findings indicate that specific domains of WAC, particularly the WW domain, dictate its colocalization with nuclear speckles, while the ability of WAC to form condensates is conferred through the CC domain, which stores and localizes client proteins such as RBM12 within nuclear speckles, allowing them to fulfill functional roles.

### 2.7 RBM12 Regulates Alternative Splicing as a Potential Component of the U5 snRNP

RBM12 is a poorly characterized factor involved in pre‐mRNA splicing. To elucidate its role, RIP‐seq was performed to identify its RNA‐binding targets. KEGG pathway analysis of the RBM12 IP group revealed an enrichment of the “spliceosome” term (Extended Data Figure 4a). Notably, transcripts of snRNAs, such as U5 snRNA, a major component of the spliceosome, were identified in the RIP‐seq data (Extended Data Figure 4b). The binding of RBM12 to U5 snRNA was further confirmed by RIP‐qPCR (Extended Data Figure 4c). These results suggest a potential interaction between RBM12 and the U5 snRNP.

As SNRNP200 (also known as BRR2 helicase) and PRPF6 are essential components of the U5 snRNP,^[^ ^25^ ^]^ we sought to validate the interaction of RBM12 with the U5 snRNP via examining these U5 snRNP components. Indeed, our co‐IP experiments confirmed the interaction of RBM12 with both SNRNP200 and PRPF6 (Extended Data Figure 4d,e). Treating cell lysates with RNase or RRI (RNase inhibitor) did not interfere with interactions between RBM12 and SNRNP200 in the CoIP experiments (Extended Data Figure 4f), suggesting that the interaction is likely to be independent of RNA.

To further investigate the role of RBM12 in alternative splicing, RNA‐seq analysis was performed upon RBM12 knockdown in cells (Extended Data Figure 5b). This analysis identified a series of alternative splicing events (ASEs) regulated by RBM12, which were characterized by significant changes in percent spliced in (PSI) values (≥0.15) (Extended Data Figure 5c). The types of ASEs regulated by RBM12 include skipped exons (SEs), alternative 5′ splice sites (A5Es), alternative 3′ splice sites (A3Es), retained introns (RIs), and mutually exclusive exons (MXEs), with SEs being the most prominent (Extended Data Figure 5d). Taken together, these results suggest that RBM12 functions as a novel component of the U5 snRNP and participates in the regulation of alternative splicing.

### 2.8 Phase Separation of WAC Influences Pre‐mRNA Splicing

**Figure 7:**
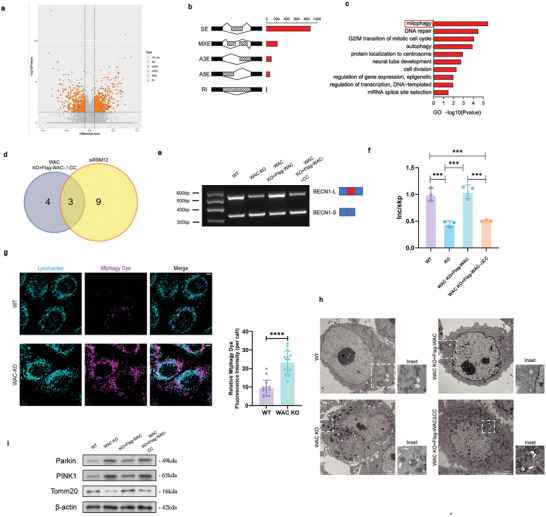
WAC‐related alternative splicing (AS) regulates mitophagy. a) Volcano plot showing the distribution of differential alternative splicing events (DASEs) between WAC knockout (KO) cells re‐expressing full‐length Flag‐WAC and WAC KO cells re‐expressing Flag‐WACΔCC. b) Quantification of DASEs between WAC KO cells re‐expressing full‐length Flag‐WAC and WAC KO cells re‐expressing Flag‐WACΔCC. c) Gene Ontology (GO) analysis of Biological Processes in WAC KO cells expressing full‐length Flag‐WAC vs. WAC KO cells expressing Flag‐WACΔCC. d) Venn diagram of the DASEs of mitophagy‐related genes in the two groups according to GO analysis (group 1: siNC vs. siRBM12; group 2: WAC‐KO WAC cells re‐expressing full‐length Flag‐WAC vs. WAC‐KO cells re‐expressing Flag‐WACΔCC). e‐f) Validation of AS events of BECN1 by semi‐quantitative RT‒PCR in wild‐type HeLa cells, WAC‐KO Cas9 cells, WAC knockout (KO) cells re‐expressing full‐length Flag‐WAC and WAC KO cells re‐expressing Flag‐WACΔCC. The relative Inc/Skip (inclusion band/exclusion band) ratio was plotted (n = 3, Student's t‐test). g) Mitophagy levels were detected by Mtphagy dye and LysoTracker in WT HeLa cells and WAC KO cells. Scale bar = 5 µm. The quantification of the mean cellular fluorescence intensity of Mtphagy Dye staining that colocalizes with LysoTracker. Cells from three independent experiments were randomly selected and quantified (n = 15, Student's t‐test). Scale bar = 5 µm. h) Representative images of mitochondria that are selectively sequestered within autophagosomes in each group. Scale bar = 2 µm. I) Immunoblotting of BECN1‐S‐associated PINK1/Parkin pathway components in wild‐type HeLa cells, WAC‐KO Cas9 cells, WAC knockout (KO) cells re‐expressing full‐length Flag‐WAC and WAC KO cells re‐expressing Flag‐WACΔCC. Beta‐actin was used as an internal control. ^*^ *p* < 0.05; ^**^ *p* < 0.01; ^***^ *p* < 0.001; ^****^ *p* < 0.0001. The results are from more than three independent experiments. Values are mean ± SD.

Given the discovery that WAC forms condensate within nuclear speckles through phase separation, we aimed to explore whether the phase separation driven by the CC domain of WAC influences pre‐mRNA splicing. To further investigate the impact of WAC condensates on splicing control, we generated stable WAC knockout (KO) cell lines expressing Flag–WAC or Flag–WACΔCC via lentiviral transfection (Extended Data Figure 5a). We identified numerous AS events regulated by the WAC CC domain that exhibited significant changes (|PSI| ≥ 0.15, P < 0.05, FDR < 0.05) (**Figure** **7**a,b). Concurrently, the regulatory effect of the WAC CC on the transcriptome was more subtle than that of AS events (Extended Data Figure 5f).

### 2.9 WAC‐Associated ASE Leads to Hyperactivated Mitophagy

**Figure 8:**
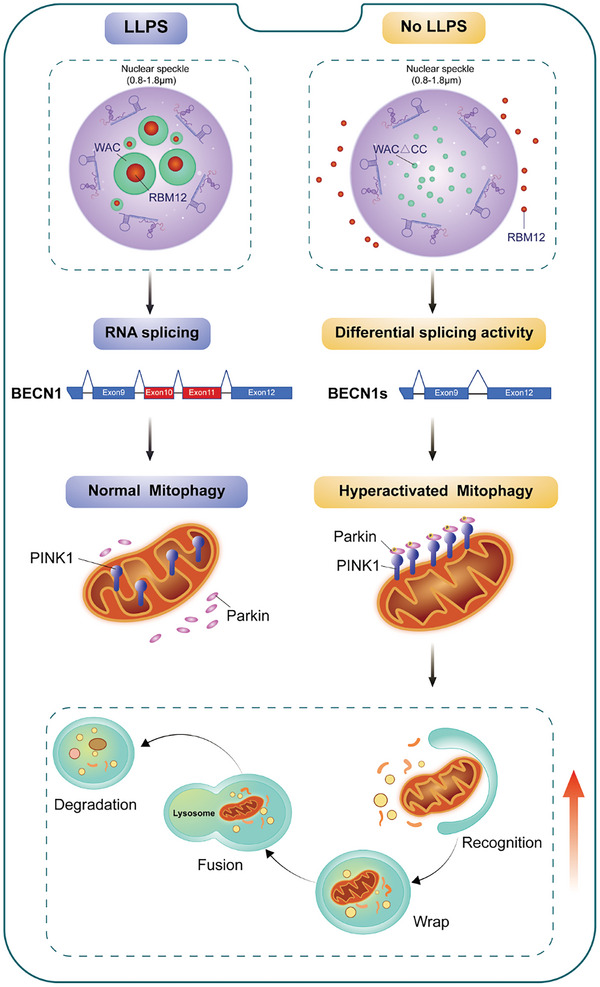
A model of enhanced mitophagy regulated by alternative splicing driven by nuclear condensation of WAC.

Given the potential role of RBM12 as a novel component of the U5 snRNP involved in pre‐mRNA splicing, we further investigated whether WAC, through its ability to form scaffold proteins through phase separation, could regulate AS by modulating the client protein RBM12. An in‐depth analysis of AS events regulated by WAC, WACΔCC, and RBM12 revealed their impact on cellular functions, notably mitochondrial autophagy, as identified through GO analysis (Extended Data Figure 5e; Figure 7c). Importantly, the key cellular functions of AS events regulated by WACΔCC overlapped with those regulated by RBM12, with a focus on genes within the mitochondrial autophagy pathway, including the BECN1 gene, which produces two splice isoforms (Extended Data Figure 5g). The BECN1 (Beclin 1) gene encodes the BECN1 protein, which plays a crucial role in autophagy. In mitochondrial autophagy, BECN1 interacts with other autophagy‐related proteins (such as Atg14L/Barkor, VPS34, and VPS15) to form a unique complex, facilitating the identification and encapsulation of dysfunctional mitochondria for degradation by autophagosomes.^[^ ^26^ ^]^ Notably, the short splice variant BECN1‐S, resulting from the loss of exons 10 and 11 in the long splice variant BECN1‐L, was detected (Extended Data Figure 5g). Semi‐quantitative PCR validated the shift from BECN1‐L to BECN1‐S in WAC‐KO Cas9 cells and cells expressing Flag–WACΔCC (Figure 7e,f). Studies have shown that direct overexpression of BECN1‐S can mediate the occurrence of mitochondrial autophagy, while knocking down BECN1‐S inhibits this process.^[^ ^27^ ^]^ Immunoblotting further confirmed that the protein levels of Parkin and PINK1 were increased, while TOMM20 levels were decreased in WAC‐KO Cas9 and Flag–WACΔCC cells (Figure 7i). Additionally, the use of Mtphagy dye, a small‐molecule fluorescent probe for visualizing mitophagy, and the lysosomal marker LysoTracker further confirmed that mitophagy levels were elevated in WAC‐KO cells (Figure 7g). The results from the transmission electron microscope (TEM) indicated that the loss of full‐length WAC and the loss of the coiled‐coil domain of WAC both lead to hyperactivated mitophagy (Figure 7h). These findings are consistent with those obtained using the Mtphagy dye. To verify that this hyperactivation was regulated by WAC‐related AS, we overexpressed BECN1‐S using a lentiviral vector. Compared to the negative control cells, cells overexpressing BECN1‐S exhibited subsequent hyperactivation of mitophagy (Extended Data Figure 6a). The mitophagy PINK1/Parkin pathway was confirmed to be upregulated in cells overexpressing BECN1‐S, while expression of the mitochondrial protein TOMM20 was downregulated (Extended Data Figure 6d). We conducted the BECN1 knockdown experiment in WAC KO cells using small interfering RNA (siRNA) and confirmed the knockdown efficiency of BECN1‐S by qRT‐PCR (Extended Data Figure 6c). By knocking down BECN1‐S in WAC KO cells, we observed attenuated mitophagy activity by Mtphagy dye (Extended Data Figure 6b) (**Figure** **8**).

## 3 Discussion

In the present study, we revealed a mechanism by which WAC forms condensates within nuclear speckles to recruit splicing‐related factors, ultimately regulating AS. This regulation, mediated by the phase separation ability of WAC, further modulates mitochondrial autophagy through RBM12 by affecting BECN1 AS. As such, our findings shed new light on potential pathophysiologic components of disordered mitophagy.

The composition and functionality of intracellular biomolecular condensates are poorly understood. Nuclear speckles play a pivotal role in mRNA processing, resulting in precise gene expression outcomes. The dynamic nature of nuclear speckles underscores their importance in the cellular architecture, with numerous proteins within these structures demonstrating the ability to form biomolecular condensates. This capacity is essential for the regulation of gene expression, highlighting a sophisticated level of cellular organization that impacts mRNA splicing and processing.

WAC, a WW domain‐containing adapter protein with a CC domain, was identified in 2002 and has garnered increased attention for its role in posttranslational modifications.^[^ ^17^ ^]^ WAC specifically recognizes the RNA polymerase II C‐terminal domain via its WW domain, orchestrating H2B ubiquitination at gene transcription sites by recruiting RNF20/40 and RAD6.^[^ ^13^ ^]^ This process underscores the complexity of cellular regulatory mechanisms, where ubiquitination not only serves as a posttranslational modification but also plays a critical role in the organization of phase‐separated structures within the cell. The intricate relationship between histone ubiquitination and AS is further exemplified by proteins such as USP49 and USP42, which, in addition to their roles in H2B ubiquitination and deubiquitination, also contribute to the regulation of AS events.^[^ ^28^, ^29^ ^]^

By exploring the phase‐separated characteristics of WAC, we revealed its propensity to form liquid droplets in the nucleus via liquid–liquid phase separation (LLPS). This discovery was facilitated by a detailed examination of the amino acid sequence of WAC, pinpointing the CC domain as a critical phase‐separation driver. Notably, the presence of five leucines within this domain appears to facilitate the formation of WAC condensates through hydrophobic interactions, suggesting a mechanism by which WAC could exert its regulatory effects within the nucleus.

Our investigation identified RBM12 as a potential client protein recruited to nuclear WAC condensates. Despite lacking inherent phase‐separated properties, the recruitment of RBM12 into these condensates and its binding to U5 snRNA hint at its involvement with the U5 snRNP, illustrating the complex interplay between phase separation, protein recruitment, and RNA processing. Furthermore, we elucidated WAC‐associated splicing events, revealing that enhanced mitophagy activity is linked to the regulatory influence of WAC. This insight into the role of WAC in RNA biogenesis, particularly within nuclear speckles, opens new avenues for understanding the multifaceted mechanisms governing gene expression.

In eukaryotes, the regulation of gene expression is a multistep and highly complex process that is meticulously balanced to maintain physiological equilibrium. Disruptions in this balance, especially through aberrant AS, can lead to a wide range of diseases, including metabolic disorders, neurodegenerative diseases, and cancer. Our findings not only reveal that the WAC protein forms a scaffold within nuclear speckles by recruiting client proteins to specific condensates through its multivalent domain but also highlight the role of condensate absence in generating BECN1‐S, thereby impacting mitochondrial autophagy. The connection between the regulation of AS and mitochondrial autophagy by WAC condensates underscores a novel pathway that could contribute to the pathogenesis of diseases related to WAC dysfunction or imbalances in mitochondrial autophagy homeostasis, offering new insights into the cellular and molecular mechanisms driving disease progression.

Patients with loss‐of‐function mutations in WAC were first identified as having Desanto‐Shinawi syndrome in 2015, which is characterized by a neurocognitive phenotype and facial dysmorphism.^[^ ^16^ ^]^ Interestingly, all patients with Desanto‐Shinawi syndrome and loss‐of‐function mutations in WAC appear to have lost the CC domain of WAC,^[^ ^30^ ^]^ which strengthens our hypothesis that phase‐separated characteristics play an important role in nuclear biological functions. These findings will contribute to our understanding of biomolecular condensates and the potential pathogenesis through which improperly regulated AS leads to Desanto‐Shinawi syndrome.

## 4 Experimental Section

### Plasmids and Antibodies

To create eukaryotic expression vectors, cDNA was amplified through a conventional PCR process. The amplified cDNA was then inserted into the pcDNA3.1 vector via a Seamless Assembly Kit (CL140, Biomed). Constructs of Cry2 featuring WAC CC domain sequences were produced by incorporating these fragments into the Lenti‐mCherry‐Cry2 framework using the Seamless Assembly Kit. To generate WAC 5LE constructs, the 5LE mutant sequence of WAC was synthesized by the company (GENEWIZ, Inc., SUZHOU). Then, the WAC fragment and 5LE fragment were amplified using a standard PCR‐based approach, and the two fragments were assembled into the pcDNA3.1 vector using a Seamless Assembly Kit.

The following antibodies were obtained from commercial sources. anti‐SON (sc‐398508; Santa Cruz Biotechnology, 1:100), anti‐SC35 (ab11826; abcam, 1:200), anti‐WAC (ab109486; abcam, 1:100 or 1:1000), anti‐RBM12 (sc‐514259; Santa Cruz Biotechnology, 1:100 or 1:1000), anti‐NPM1 (FC‐61991; Thermo, 1:200), anti‐Coilin (ab11822; abcam, 1:200), Donkey Anti‐Rabbit IgG H&L (Alexa Fluor 647) (ab150075, abcam, 1:200), Donkey Anti‐Mouse IgG H&L (Alexa Fluor 555) (ab150106, abcam, 1:200), anti‐β‐actin (200068‐8F10; ZEN‐BIO, 1:2000), anti‐SC35 (ab204916; abcam, 1:1000), anti‐PINK1 (507131; ZEN‐BIO, 1:1000), anti‐Parkin (381626; ZEN‐BIO, 1:1000), anti‐TOMM20 (R25952; ZEN‐BIO, 1:2000), Goat anti mouse IgG (H&L) (HRP conjugated) (511103; ZEN‐BIO, 1:10000), Goat anti‐rabbit IgG (H&L) (HRP conjugated) (511203, ZEN‐BIO, 1:10000), anti‐Flag (Sigma, F3165, M2,1:1000), anti‐HA antibodies (H9658; Sigma, 1:1000), anti‐snRNP200 (ab241589; 1:2000), and anti‐WAC (Merck; ABE471, Merck, 1:1000).

### Cell Culture

HeLa and HEK293T cells obtained from Shanghai Model Organisms (www.modelorg.com) were cultured in Dulbecco's modified Eagle's medium (DMEM, Gibco) supplemented with 10% fetal bovine serum (Gibco) and 1% penicillin/streptomycin (Thermo Fisher Scientific). The cells were routinely maintained in a humidified atmosphere at 37 °C in a CO_2_ incubator (Thermo Fisher Scientific).

### Plasmid Transfection

Plasmid transfection was performed using Lipofectamine 3000 (Invitrogen) according to the manufacturer's instructions. The cells were used in experiments 24–72 h after transfection.

### Lentivirus Transfection and siRNA Interference

The lentiviral vectors Lv‐WAC and Lv‐WACΔCC with mCherry and the lentiviral vectors Lv‐BECN1‐S and Lv‐NC with GFP were obtained from GeneChem (Shanghai, www.genechem.com.cn). Briefly, lentiviral vectors were transfected into cells according to the manufacturer's instructions. The cells were used in experiments 48 h after transfection.

For siRNA transfection, cells were plated and subjected to siRNA (50 nM) transfection with Lipofectamine RNAiMAX (Invitrogen) for 6 h. After replacing with a growth medium without antibiotics, cells were cultured for another 24–48 h for the following experiments.

### Immunofluorescence and Colocalization Assessment

Immunofluorescence was performed as described previously.^[^ ^31^ ^]^ Treated cells were fixed in 4% paraformaldehyde for 15 min at room temperature and permeabilized with 0.25% Triton X‐100. Subsequently, the samples were blocked with 5% bovine serum albumin (BSA) for 1 h. The membranes were incubated overnight at 4 °C with the primary antibody, followed by incubation with the secondary antibody for 2 h at room temperature. 4′,6‐diamidino‐2‐phenylindole (DAPI) was used for nuclear visualization. Images were acquired with an Olympus FV3000 confocal laser scanning microscope and an Olympus SpinSR Spinning Disk Confocal microscope. Images were analyzed with ImageJ.

### Immunoblotting

Proteins were harvested with RIPA buffer supplemented with 1% phenylmethylsulfonyl fluoride (PMSF; ST507; Beyotime). Isolated proteins on SDS‒PAGE gels (PG112, Epizyme) were transferred onto polyvinylidene fluoride membranes (IPVH00010; Millipore). The membranes were blocked with 5% skim milk, and then incubated with primary antibodies overnight. Subsequently, the membranes were incubated with secondary antibodies at room temperature for 2 h before visualization with Super ECL Detection Reagent (36208ES, Yeasen).

### Coimmunoprecipitation (CoIP)

CoIP experiments with plasmid transfection were conducted in HEK293T cells, while CoIP experiments with endogenous proteins were performed in HeLa cells. Cells were lysed using an appropriate lysis buffer, and the resulting cell lysates were clarified prior to immunoprecipitation with anti‐Flag M2 agarose beads (Sigma). Following immunoprecipitation, protein complexes were isolated, resolved by SDS‒PAGE, and subjected to western blotting for the detection of specific proteins with anti‐HA antibodies. For endogenous protein CoIP, protein extracts were incubated with anti‐snRNP200, anti‐WAC or control IgG from the same species overnight at 4 °C. The immune complexes were then bound to Pierce Protein A/G magnetic beads (Thermo Fisher Scientific, Inc.). After the supernatant was removed by centrifugation, the eluted proteins were processed for immunoblotting analysis. Co‐immunoprecipitation with or without RNase treatment was done as follows: The cell lysates were treated with RNase I (Ambion) at 37 °C for 10 min or treated with RiboLock RNase Inhibitor (Thermo Scientific, Cat. No. EO0381) for 10 min at room temperature before proceeding with the following co‐immunoprecipitation protocol.

### Protein Purification

The HEK293T expression system was used to express recombinant proteins. 48 h after plasmid transfection, the cells were collected, washed, resuspended in harvest buffer (50 mm Tris, pH 8.0, 150 mm KCl), and frozen in liquid nitrogen. Lysates were clarified by centrifugation at 10000 × g for 10 min. Afterward, the supernatants were incubated with anti‐DYKDDDDK G1 Affinity (GenScript; L00432‐10) for 3 h at 4 °C. The beads were washed 3 times with lysis buffer and proteins were eluted with 50 mm Tris, pH 8.0, 150 mm NaCl, and 250 µg mL^−1^ FLAG peptide (MCE 98849‐88‐8) buffer. The proteins were stored at −80 °C.

### Blue Light Treatment

The indicated Cry2‐mCherry‐fusion plasmids were transfected into cells, activated by blue light, and the samples were observed by time‐lapse confocal microscopy. The cells were subjected to continuous blue light stimulation using an Olympus FV3000 confocal laser scanning microscope.

### CRISPR/Cas9‐Mediated Knockout of the WAC Gene in HeLa Cells

CRISPR/Cas9 was employed to target exon 3 of the WAC gene using a guide RNA (5′‐ATTTGGTGGTGAAGGATCTC‐3′) cloned alongside Cas9 and a puromycin resistance cassette within a vector, which also included a GFP reporter. Donor DNA with 800 bp homology arms flanking the single guide RNA target site and FRT sites was used for homology directed repair. Transfection was performed according to the manufacturer's protocol for the selected reagents. Posttransfection, puromycin selection, and GFP expression facilitated the isolation of successful knockouts. Clones were single‐cell sorted, PCR screened, and validated by Sanger sequencing. Positive clones were expanded for further analysis.

### Fluorescence Recovery after Photobleaching (FRAP)

FRAP analysis was carried out on an Olympus FV3000 confocal laser scanning microscope. After 3 prebleach frames, the fluorescence signal from a region of interest was bleached with a 402 nm laser at 10% power. EasyFRAP software (https://easyfrap.vmnet.upatras.gr/) was used to rectify and normalize the data.

### In Vitro Droplet‐Formation Assay

Quantification of droplet size from in vitro droplet‐formation assays was performed with ImageJ. Briefly, images were converted to 8‐bit format, and the threshold was adjusted based on the intensity. The detected objects in each image were examined manually to confirm their validity.

### RNA FISH and Immunofluorescence

RNA FISH was performed as previously described.^[^ ^32^ ^]^ The probes used for U2 detection were single‐stranded DNA oligos labeled with Cy3 (5′‐AACAGATACTACACTTGATCTTAGCCAAAAGGCCGAGAAGC‐3′, Shenggong, Shanghai). The cells were fixed in 4% paraformaldehyde for 10 min and then permeabilized in 70% ethanol overnight. A labeled oligo probe was added to the hybridization buffer. Hybridization was performed in a humidified chamber at 37 °C overnight. For combined RNA FISH/immunostaining, RNA FISH was performed first, followed by immunofluorescence staining as described above.

### Detection of Mitophagy Levels

Mtphagy Dye (Dojindo) and LysoTracker Blue DND‐22 (Invitrogen) were used to assess cellular mitophagy levels and confirm the fusion of Mtphagy Dye‐labeled mitochondria and lysosomes. Briefly, cells were cultured in confocal dishes. An appropriate volume of 100 nm Mtphagy Dye working solution was added to the cells, which were then incubated at 37 °C for 30 min. An appropriate volume of 50 µm CCCP (Solarbio) was added to the cell medium overnight to activate mitophagy. Before observation by confocal laser scanning microscopy, the lysosomes were labeled with 50 nm of LysoTracker Blue DND‐22.

### Immunoprecipitation‐Mass Spectrometry (IP‐MS)

HEK293T cells transfected with Flag‐eGFP‐WAC and Flag‐eGFP were lysed with immunoprecipitation lysis buffer. The cell lysates were subjected to immunoprecipitation with anti‐DYKDDDDK G1 Affinity Resin (L00432, GenScript). The candidate proteins interacting with eGFP‐WAC were identified by 3D mass spectrometry and supported by PTM bio, Hangzhou, China (http://www.ptm‐biolab.com.cn).

### RNA‐Seq

After siRNA interference or lentivirus transfection for 48–72 h, cell lysates were collected with TRIzol (15596026, Thermo Fisher Scientific). The RNA‐seq experiment and high‐throughput sequencing, as well as the data analysis, were supported by Seqhealth Technology Co., Ltd., Wuhan, China (http://www.seqhealth.cn).

### RT‒qPCR

Total RNA was obtained using TRIzol (15596026, Thermo Fisher Scientific) as previously described.^[^ ^33^ ^]^ cDNA synthesis was performed with a Hifair III 1st Strand cDNA Synthesis Kit (11139; Yeasen). Real‐time amplification was detected with Hieff UNICON Universal Blue qPCR SYBR Master Mix (11184, Yeasen) on a CFX96 instrument (Bio‐Rad). GAPDH was used as an internal control, and relative target gene expression was evaluated with the 2‐ΔΔCt method.

### RIP‐Seq and RIP‐qPCR

The cells were treated with cell lysis buffer. The 10% lysate sample was stored and named “Input”, 80% was used in immunoprecipitation reactions with an anti‐RBM12 antibody (sc‐514259; Santa Cruz Biotechnology) and named “IP”, and 10% was incubated with normal mouse IgG (12‐371, Sigma) as a negative control and named “IgG”. The RNA for input and IP was extracted using TRIzol reagent (15596026, Thermo Fisher Scientific). The library preparation and high‐throughput sequencing were supported by Seqhealth Technology Co., Ltd., Wuhan, China (http://www.seqhealth.cn). For RIP‐qPCR, the RNAs were quantified through RT‒qPCR.

### RT‒PCR

Total RNA was obtained using TRIzol (15596026, Thermo Fisher Scientific). cDNA synthesis was performed with a Hifair III 1st Strand cDNA Synthesis Kit (11139; Yeasen). RT‒PCR was performed using 2×Hieff UNICON HotStart PCR Master Mix (10732; Yeasen). The PCR was carried out on a Mastercycler (Eppendorf) and consisted of an initial denaturation at 95 °C for 5 min, followed by a cycle program of 95 °C for 30 s, 55 °C for 30 s, 72 °C for 30 s, and a final extension at 72 °C for 10min. The PCR consisted of 35–40 cycles. The PCR products were resolved on 2% agarose gel mixed with Ultra GelRed (GR501, Vazyme). The relative band intensity was determined using ImageJ.

### Transmission Electron Microscope

After transfected with siRNA or lentivirus, cells were washed, trypsinized, and fixed with 2.5% Glutaraldehyde (Solarbio). A Transmission electron microscope (JEM‐1400FLASH) was utilized to detect mitochondria that were selectively sequestered within double‐membrane vesicles at the Lilai biomedicine experiment center (Chengdu, China)

### Statistical Analysis

All the experiments were tested at least three biological replicates. All analyses were performed using GraphPad Prism version 8.0 (GraphPad Software). Data were presented as mean ± SD. Statistical significance was assessed using Student's t‐test, one‐way ANOVA, or two‐way ANOVA, as appropriate. A p‐value of less than 0.05 was considered statistically significant and denoted with an asterisk (“^*^”). P‐values less than 0.01, 0.001, and 0.0001 were denoted with two asterisks (“^**^”), three asterisks (“^***^”), and four asterisks (“^****^”), respectively.

## Conflict of Interest

The authors declare no conflict of interest.

## Author Contributions

J.W. and Y.F. contributed equally to this work. The conceptualization was led by C.C.Z. and Z.W.Z.; the methodology was developed by J.H.W., C.C.Z., Z.W.Z., L.J.W., L.X., G.W.L., Z.X.W., J.Y.W., and Z.Y.P.; the investigation was conducted by J.H.W., C.C.Z., Z.W.Z., L.J.W., L.X., and J.Y.W.; visualization efforts were contributed by J.H.W., C.C.Z., J.Y.W., and J.J.J.; funding acquisition was handled by C.C.Z., Y.F., and Z.W.Z.; project administration was overseen by C.C.Z., Z.W.Z., Y.F., and J.H.W.; supervision was provided by C.C.Z., Z.W.Z., Y.F., Q.Y., K.F.L., and C.J.R.; the original draft was written by C.C.Z., Z.W.Z., J.H.W., K.F.L., C.J.R., and J.J.J.; and the review and editing process was carried out by C.C.Z., Z.W.Z., J.H.W., K.F.L., C.J.R., Q.Y., and L.X. The order of authorship reflects their contributions to data collection and manuscript preparation.

## Supporting information



Supporting Information

Supplemental Video 1

Supplemental Video 2

## Data Availability

The data that support the findings of this study are available from the corresponding author upon reasonable request.
