# Supplementary material for: Nuclear Condensates of WW Domain‐Containing Adaptor With Coiled‐Coil Regulate Mitophagy via Alternative Splicing
Source: Adv Sci (Weinh). 2025 Jan 22;12(10):2406759. doi: 10.1002/advs.202406759 (PMC11904943; doi:10.1002/advs.202406759)
Supplement: Supplementary file 1 — Supporting Information [file ADVS-12-2406759-s002.docx]

**Extended Data Fig.1**

**
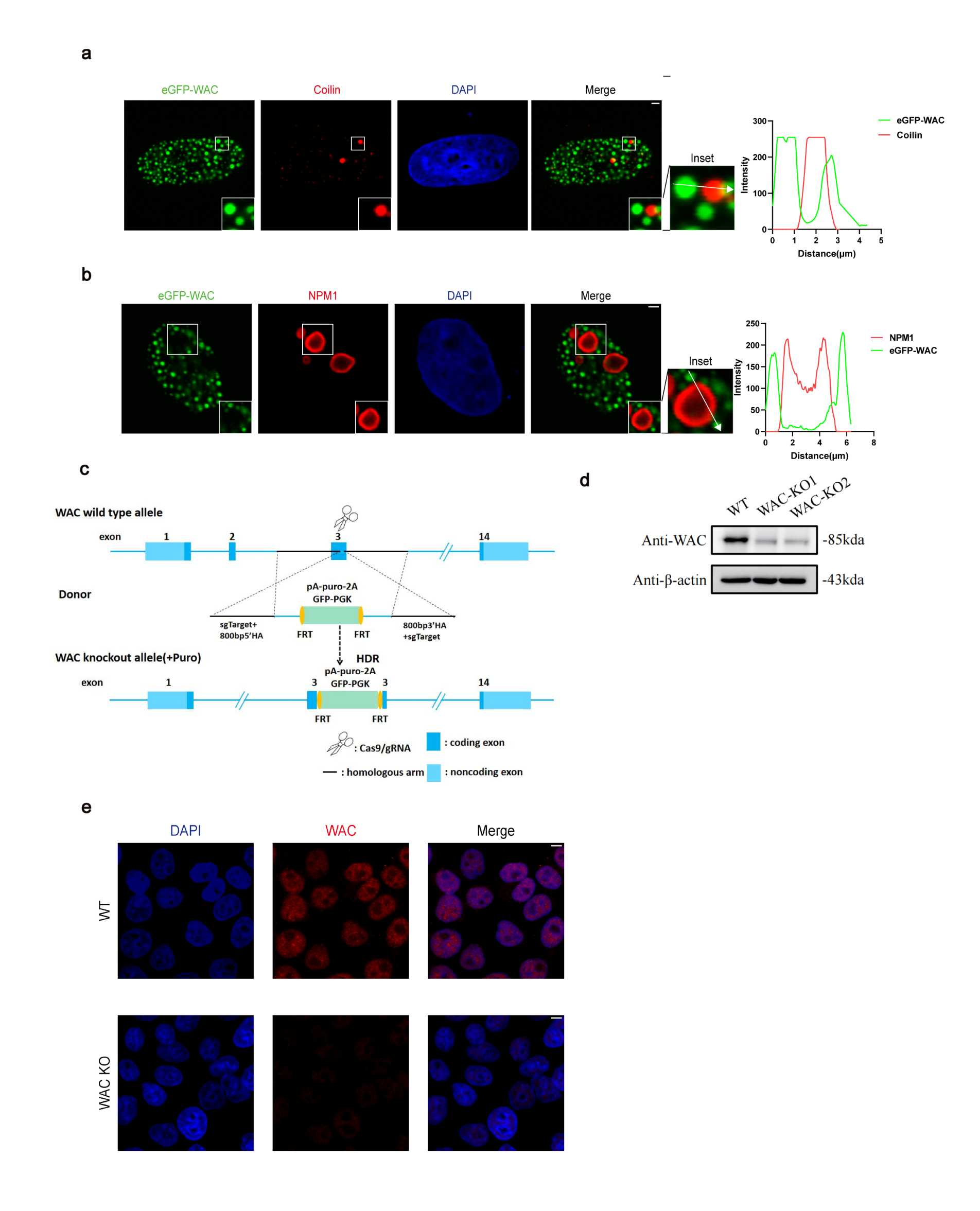
**

**Extended Data Fig. 1 | WAC nuclear condensates do not colocalize with Cajal bodies or nucleoli and regulate the expression of related genes.**

(**a-b**). Immunofluorescence images of HeLa cells transfected with full-length WAC with eGFP and endogenous NPM1 and coilin. Nuclei were stained with 4’,6-diamidino-2-phenylindole (DAPI). Scale bar=10 μm.

(**c**). The experimental workflow for the generation of the WAC knockout HeLa

cell line is shown schematically.

(**d**). The efficiency of WAC depletion in the indicated stable HeLa cell lines was determined by immunoblotting. Beta-actin expression was used as an internal control.

(**e**). The efficiency of WAC depletion in the indicated stable HeLa cell lines was determined by immunofluorescence.

**Extended Data Fig.2**


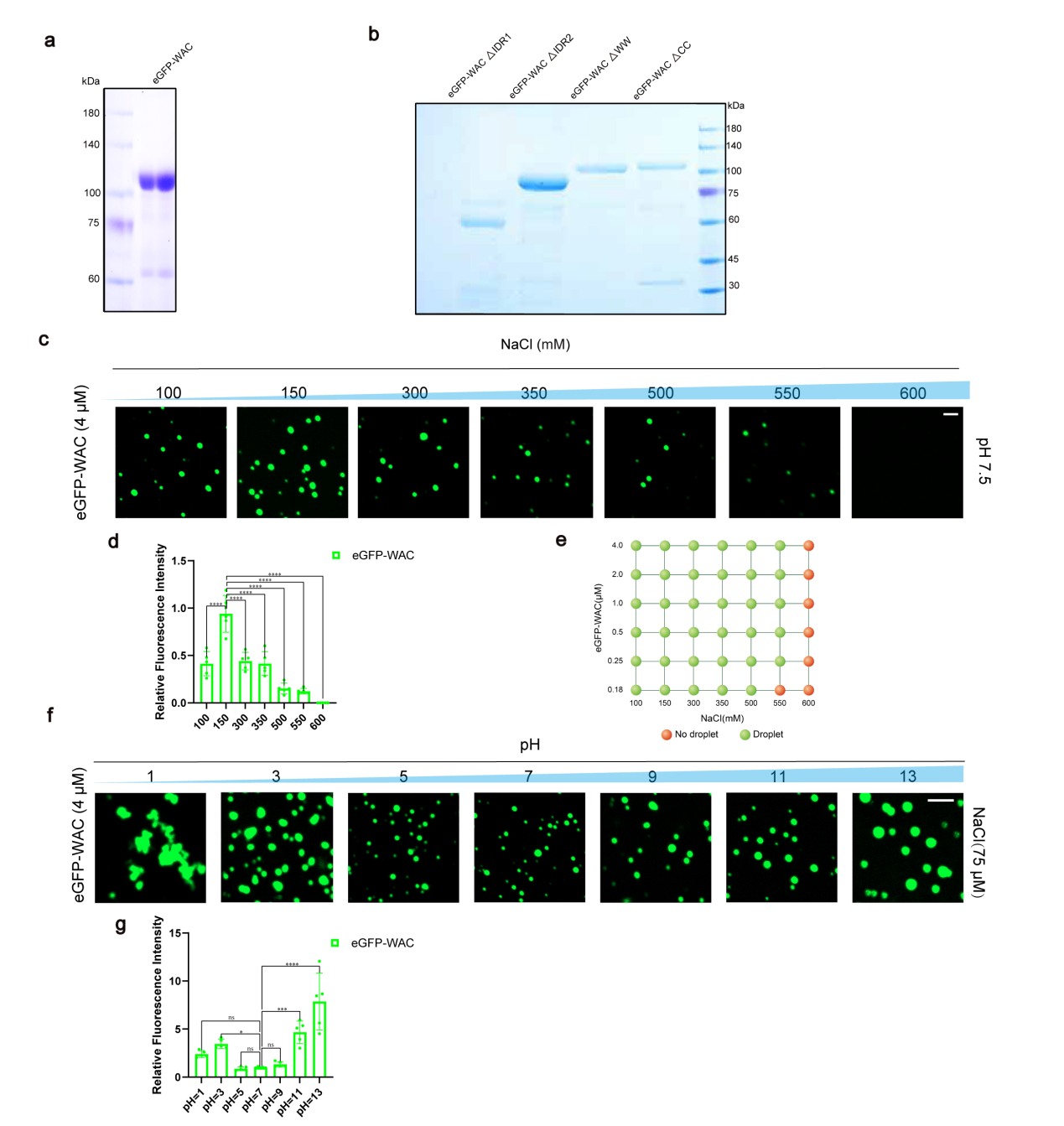


**Extended Data Fig. 2 | In vitro phase separation behavior of WAC protein under varying pH and NaCl concentrations**

(**a-b**) Coomassie blue staining of SDS‒PAGE gel showing the purity of 4 truncated versions of WAC fused eGFP.

(**c-d**). In vitro condensate formation of purified full-length WAC fusion proteins at various NaCl concentrations (100, 150, 300, 350, 500, 550, and 600 mM). The quantification of the droplets shown in Fig. S2C. Droplets in each group were quantified (n=5, Student's t-test). Scale bar=5 μm.

(**e**). Phase diagram of Figure S2C.

(**f-g**). Quantification of relative fluorescence intensity at various pH values (pH=1, 3, 5, 7, 9, 11, and 13). Droplets in each group were quantified (n=5, Student's t-test). Scale bar=5 μm.

*, p<0.05; **, p<0.01; ***, p<0.001; ****, p<0.0001. The results are from more than three independent experiments. Values are mean±SD.


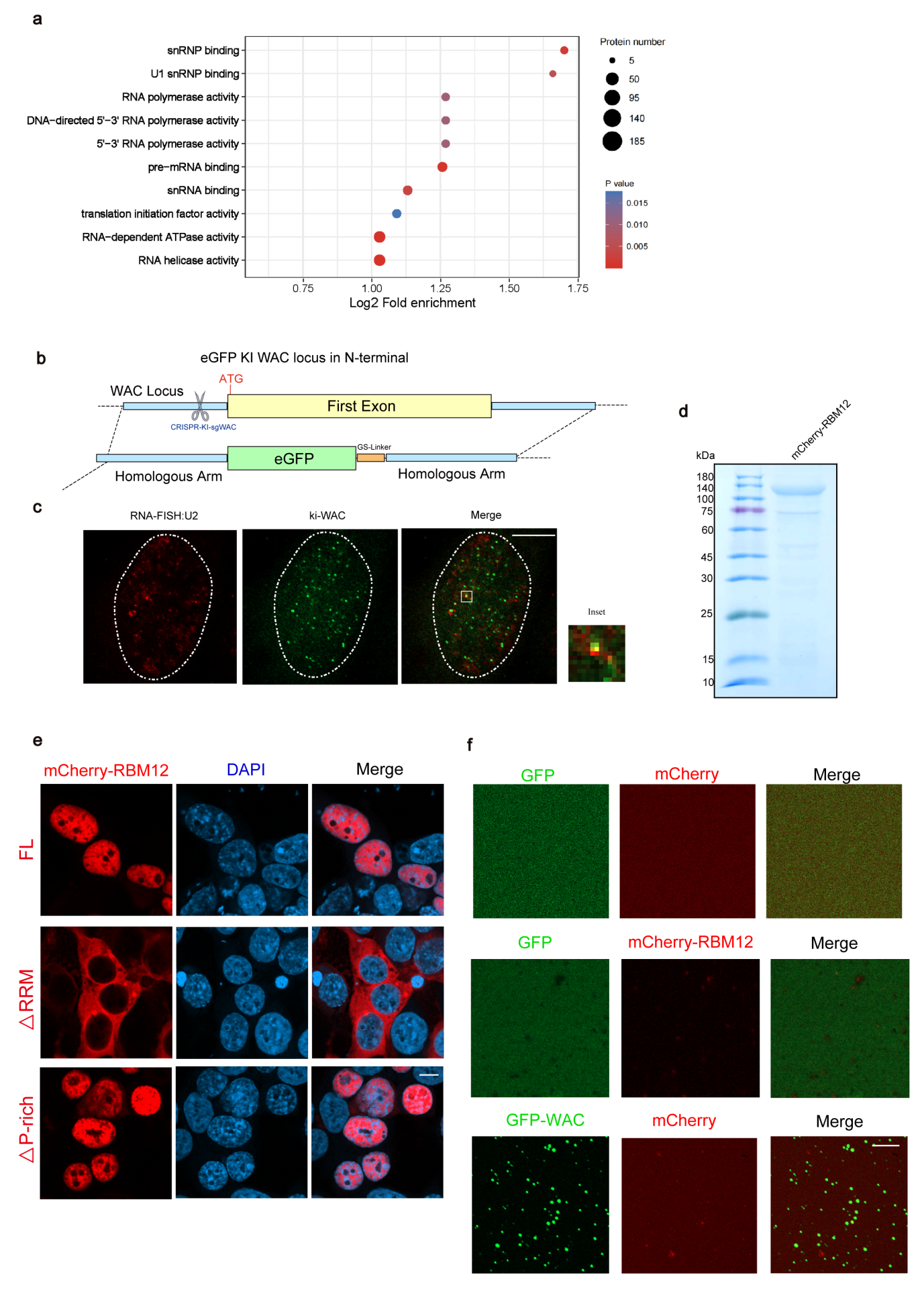
**Extended Data Fig.3**

**Extended Data Fig. 3 | Colocalization of the WAC protein with U2 snRNA, a key spliceosome component**

(**a**). Gene Ontology (GO) pathway enrichment analysis of the molecular functions of the upregulated proteins according to LC‒MS/MS analysis in the eGFP-WAC group.

(**b**). Schematic of the experimental design of WAC-EGFP knock-in by the CRISPR/Cas9 system.

(**c**). Images of the C-terminal-tagged eGFP-WAC knock-in cell line and Cy3-labeled probes for the spliceosomal marker U2 snRNA in RNA FISH.

(**d**). Coomassie blue staining of SDS‒PAGE gel showing the purity of RBM12 fused to mCherry.

(**e**). Images of HeLa cells transfected with truncated mCherry-RBM12 (ΔRRM and ΔP-rich) plasmids and transfected into cells. Nuclei were stained with 4’,6-diamidino-2-phenylindole (DAPI). Scale bar=10μm.

(**f**). In vitro condensate formation of purified GFP and mCherry, GFP and mCherry-RBM12, and eGFP-WAC and mCherry. Scale bar=5μm.


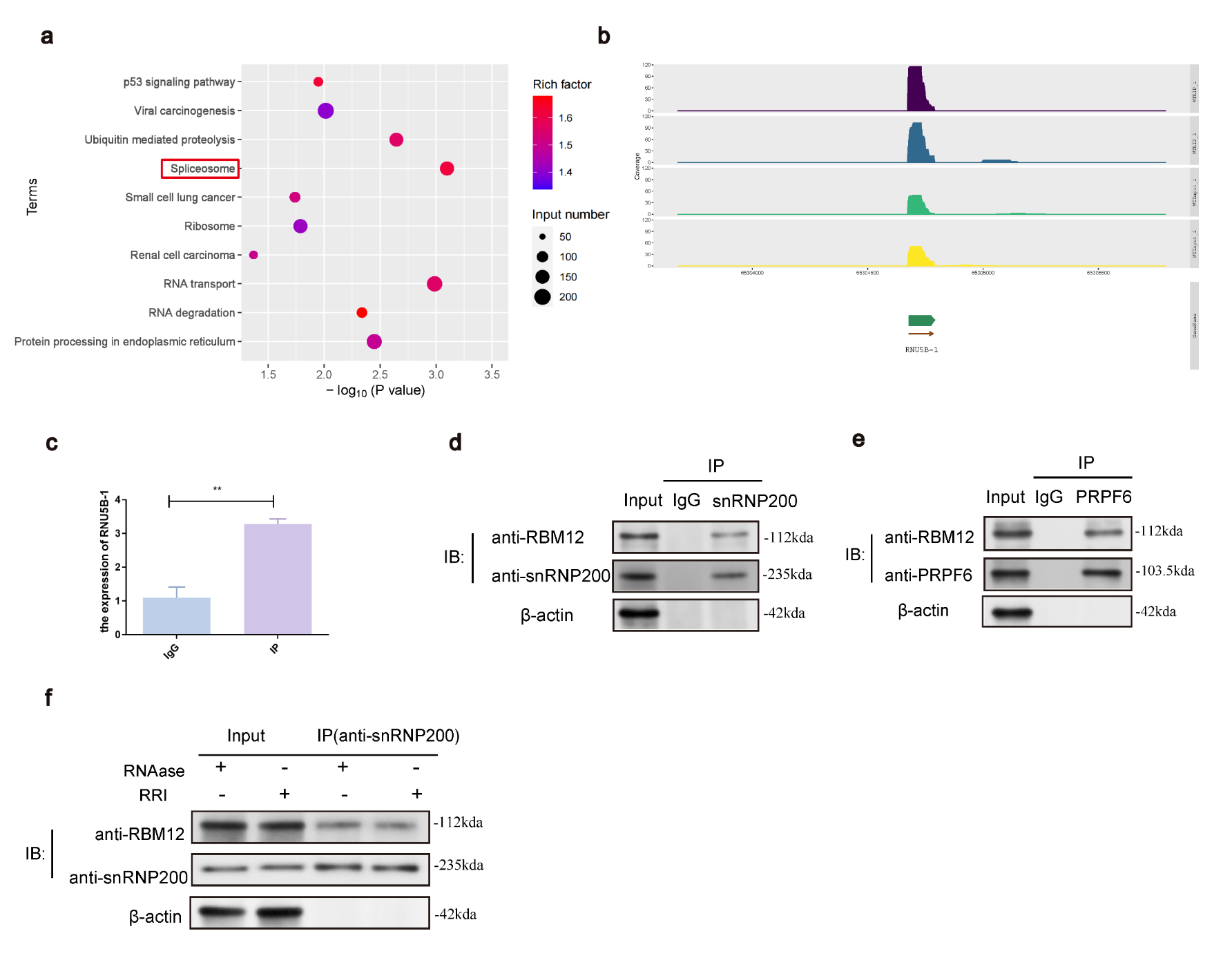
**Extended Data Fig.4**

**Extended Data Fig. 4 | RBM12 interacts with U5snRNA or the U5 snRNP subunit snRNP200.**

(**a**). Kegg pathway analysis using the KEGG database of RBM12-bound peaks in RIP-seq.

(**b**). RBM12 binds to transcripts of U5 snRNA genes.

(**c**). RIP-qPCR was performed to detect RNU5B-1 in HeLa cells by using IgG or RBM12 antibodies (n=3, Student's t-test). The data are presented as the means ± SD of three replicates.

**(d)**. Coimmunoprecipitation assay with anti-SNRNP200 antibodies for immunoprecipitation (IP), followed by western blotting with anti-RBM12 antibodies (top). The abundance of these proteins in the cell lysates was examined using western blotting (bottom).

**(e)**. Coimmunoprecipitation assay with anti-PRPF6 antibodies for immunoprecipitation (IP), followed by western blotting with anti-RBM12 antibodies (top). The abundance of these proteins in the cell lysates was examined using western blotting (bottom).

**(f).** Coimmunoprecipitation assay with anti-SNRNP200 antibodies for immunoprecipitation (IP), followed by western blotting with anti-RBM12 antibodies (top). The cell lysates were treated with RNase or RRI (RNase inhibitor). The abundance of these proteins in the cell lysates was examined using western blotting (bottom).

*, p<0.05; **, p<0.01; ***, p<0.001; ****, p<0.0001. The results are from more than three independent experiments. Values are mean±SD.


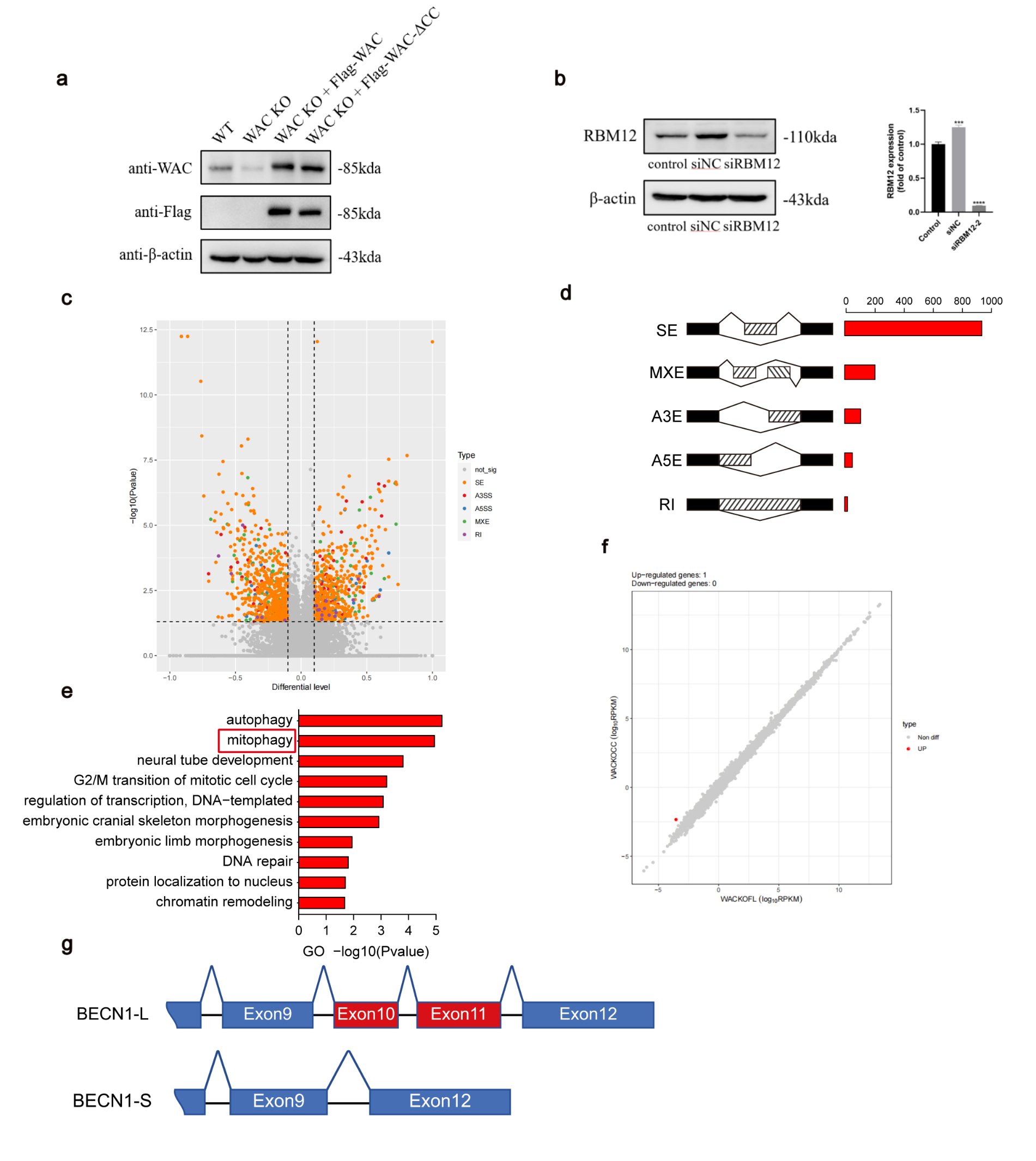
**Extended Data Fig.5**

**Extended Data** **Fig. 5 | The splicing-related factor RBM12 regulates alternative splicing events of mRNAs**

(**a**). Immunoblotting confirming the efficiency of re-expressing full-length Flag-WAC and Flag-WACΔCC in WAC knockout (KO) cells. Beta-actin was used as an internal control.

(**b**). Immunoblotting confirming the knockdown efficiency of RBM12 in HeLa cells. Beta-actin was used as an internal control. RT-qPCR confirming the knockdown efficiency of RBM12 in HeLa cells. GAPDH was used as an internal control. Data were derived from three independent experiments (n=3, Student's t-test). The data are presented as the means ± SD of three replicates.

(**c**). Volcano plot showing the distribution of differential alternative splicing events (DASEs) between siNC and siRBM12.

(**d**). Quantification of DASEs affected by RBM12.

(**e**). Gene Ontology (GO) analysis of biological processes in siNC vs. siRBM12.

(**f**). Scatter plot of expression levels between WAC KO cells re-expressing full-length Flag-WAC and WAC KO cells re-expressing Flag-WACΔCC. (annotated with differentially expressed genes).

(**g**). A schematic illustration of the cDNA sequences of BECN1-L and BECN1-S.

*, p<0.05; **, p<0.01; ***, p<0.001; ****, p<0.0001. The results are from more than three independent experiments. Values are mean±SD.

**Extended Data Fig.6**


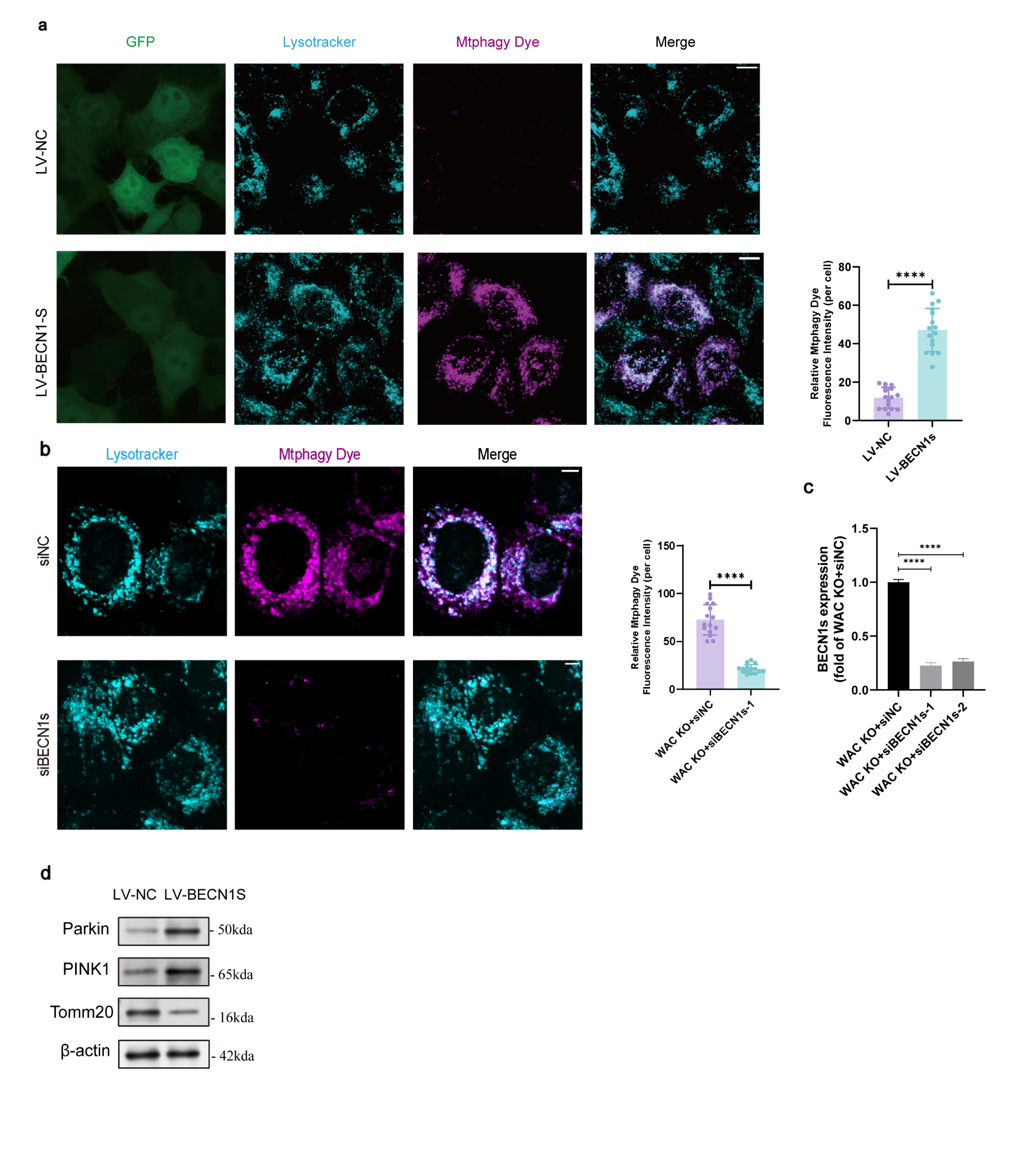


**Extended Data Fig. 6 | BECN1-S affects mitochondrial autophagy homeostasis**

(**a**). Mitophagy levels detected in HeLa cells expressing LV-NC or LV-BECN1-S using Mtphagy dye and LysoTracker. The quantification of the mean cellular fluorescence intensity of Mtphagy Dye staining that colocalize with LysoTracker. Cells from three independent experiments were randomly selected and quantified (n=15, Student's t-test). Scale bar=5μm.

(**b**). Mitophagy levels detected in WAC KO HeLa cells transfected with siNC and si-BECN1-S using Mtphagy dye and LysoTracker. Scale bar=5 μm. The quantification of the mean cellular fluorescence intensity of Mtphagy Dye staining that colocalize with LysoTracker. Cells from three independent experiments were randomly selected and quantified (n=15, Student's t-test). Scale bar=5μm.

(**c**). RT-qPCR confirming the knockdown efficiency of BECN1-S in WAC KO HeLa cells. GAPDH was used as an internal control. (n=3, Student's t-test). The data are presented as the means ± SD of three replicates.

(**d**). Immunoblotting of BECN1-associated PINK1/Parkin pathway components in HeLa cells expressing LV-NC or LV-BECN1-S. Beta-actin was used as an internal control.

*, p<0.05; **, p<0.01; ***, p<0.001; ****, p<0.0001. The results are from more than three independent experiments. Values are mean±SD.
